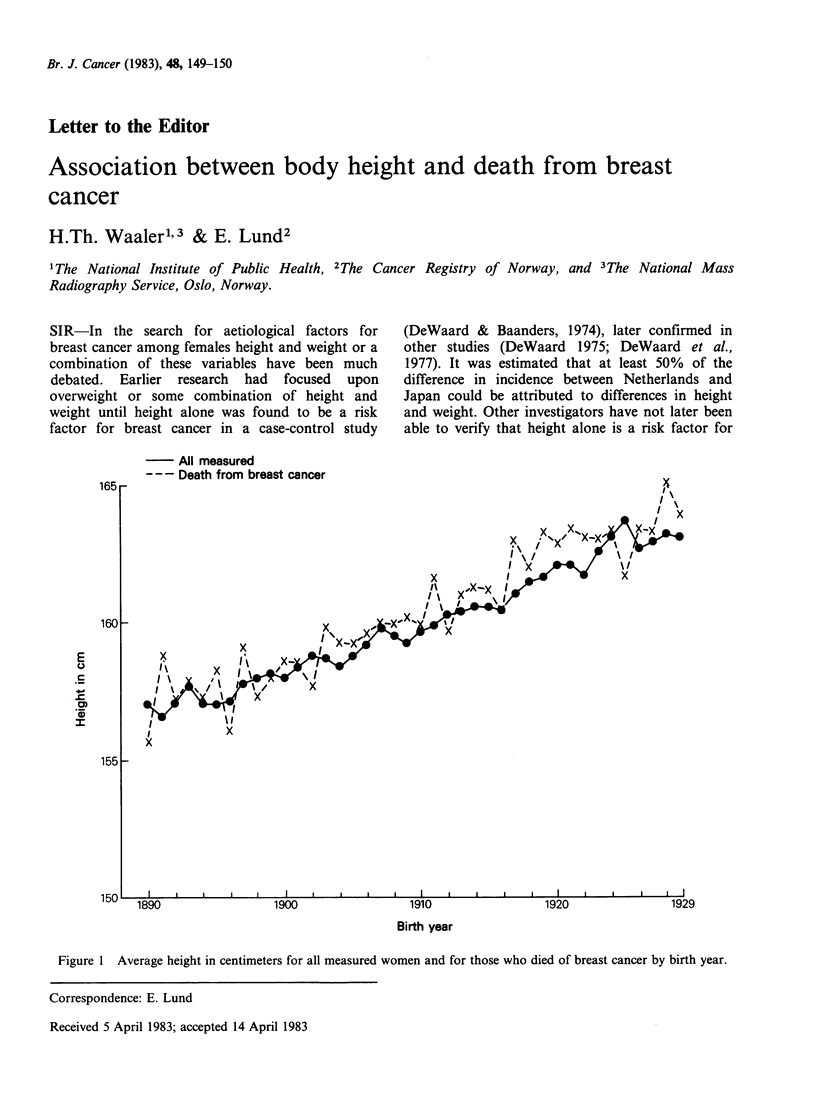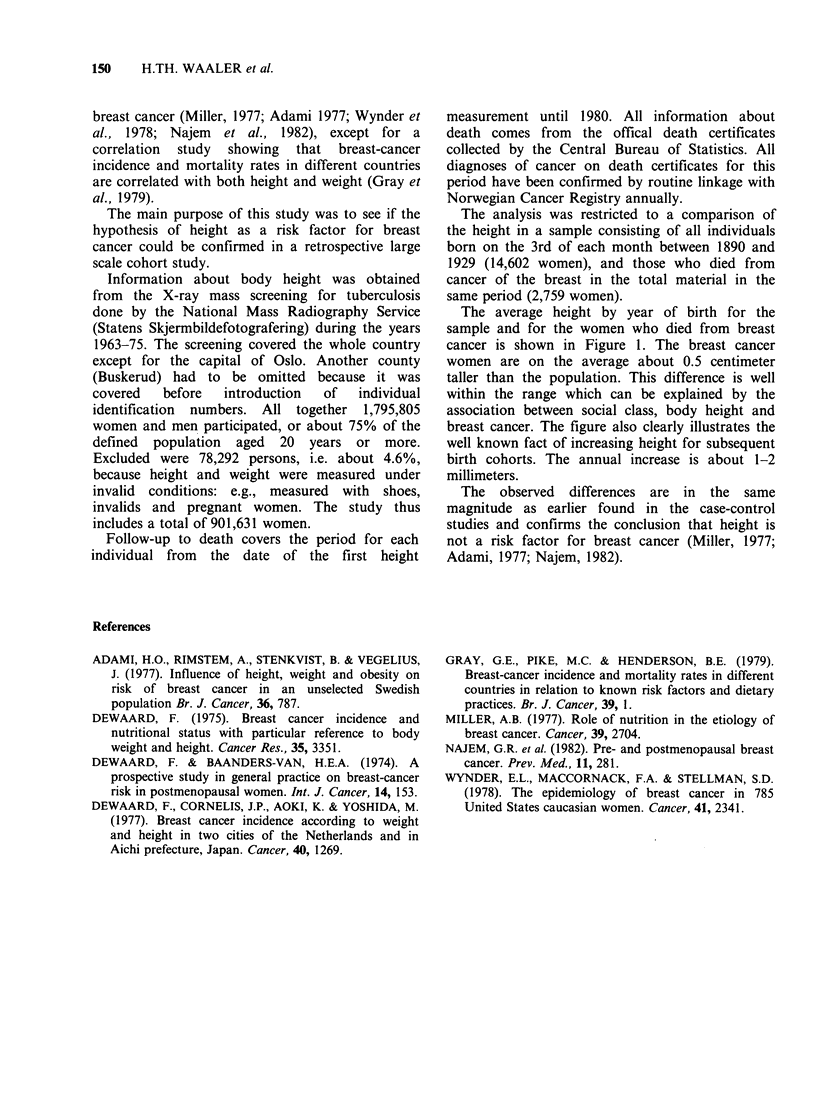# Association between body height and death from breast cancer.

**DOI:** 10.1038/bjc.1983.167

**Published:** 1983-07

**Authors:** H. T. Waaler, E. Lund


*Br. J. Cancer* (1983), **48**, 149–150

### Letter to the Editor

### Association between body height and death from breast cancer

H.Th. Waaler[1,3] & E. Lund[2]

[1]*The National Institute of Public Health,* [2]*The Cancer Registry of Norway, and* [3]*The National Mass Radiography Service, Oslo, Norway.*

SIR—In the search for aetiological factors for breast cancer among females height and weight or a combination of these variables have been much debated. Earlier research had focused upon overweight or some combination of height and weight until height alone was found to be a risk factor for breast cancer in a case-control study (DeWaard & Baanders, 1974), later confirmed in other studies (DeWaard 1975; DeWaard *et al.*, 1977). It was estimated that at least 50% of the difference in incidence between Netherlands and Japan could be attributed to differences in height and weight. Other investigators have not later been able to verify that height alone is a risk factor for

Figure 1 Average height in centimeters for all measured women and for those who died of breast cancer by birth year.

Correspondence: E. Lund

Received 5 April 1983; accepted 14 April 1983

breast cancer (Miller, 1977; Adami 1977; Wynder *et al.*, 1978; Najem *et al.*, 1982), except for a correlation study showing that breast-cancer incidence and mortality rates in different countries are correlated with both height and weight (Gray *et al.*, 1979).

The main purpose of this study was to see if the hypothesis of height as a risk factor for breast cancer could be confirmed in a retrospective large scale cohort study.

Information about body height was obtained from the X-ray mass screening for tuberculosis done by the National Mass Radiography Service (Statens Skjermbildefotografering) during the years 1963–75. The screening covered the whole country except for the capital of Oslo. Another county (Buskerud) had to be omitted because it was covered before introduction of individual identification numbers. All together 1,795,805 women and men participated, or about 75% of the defined population aged 20 years or more. Excluded were 78,292 persons, i.e. about 4.6%, because height and weight were measured under invalid conditions: e.g., measured with shoes, invalids and pregnant women. The study thus includes a total of 901,631 women.

Follow-up to death covers the period for each individual from the date of the first height measurement until 1980. All information about death comes from the offical death certificates collected by the Central Bureau of Statistics. All diagnoses of cancer on death certificates for this period have been confirmed by routine linkage with Norwegian Cancer Registry annually.

The analysis was restricted to a comparison of the height in a sample consisting of all individuals born on the 3rd of each month between 1890 and 1929 (14,602 women), and those who died from cancer of the breast in the total material in the same period (2,759 women).

The average height by year of birth for the sample and for the women who died from breast cancer is shown in Figure 1. The breast cancer women are on the average about 0.5 centimeter taller than the population. This difference is well within the range which can be explained by the association between social class, body height and breast cancer. The figure also clearly illustrates the well known fact of increasing height for subsequent birth cohorts. The annual increase is about 1–2 millimeters.

The observed differences are in the same magnitude as earlier found in the case-control studies and confirms the conclusion that height is not a risk factor for breast cancer (Miller, 1977; Adami, 1977; Najem, 1982).

### References

ADAMI, H.O., RIMSTEM, A., STENKVIST, B. & VEGELIUS, J. (1977). Influence of height, weight and obesity on risk of breast cancer in an unselected Swedish population *Br. J. Cancer*, **36**, 787.

DEWAARD, F. (1975). Breast cancer incidence and nutritional status with particular reference to body weight and height. *Cancer Res.*, **35**, 3351.

DEWAARD, F. & BAANDERS-VAN, H.E.A. (1974). A prospective study in general practice on breast-cancer risk in postmenopausal women. *Int. J. Cancer*, **14**, 153.

DEWAARD, F., CORNELIS, J.P., AOKI, K. & YOSHIDA, M. (1977). Breast cancer incidence according to weight and height in two cities of the Netherlands and in Aichi prefecture, Japan. *Cancer*, **40**, 1269.

GRAY, G.E., PIKE, M.C. & HENDERSON, B.E. (1979). Breast-cancer incidence and mortality rates in different countries in relation to known risk factors and dietary practices. *Br. J. Cancer*, **39**, 1.

MILLER, A.B. (1977). Role of nutrition in the etiology of breast cancer. *Cancer*, **39**, 2704.

NAJEM, G.R. *et al.* (1982). Pre- and postmenopausal breast cancer. *Prev. Med.*, **11**, 281.

WYNDER, E.L., MACCORNACK, F.A. & STELLMAN, S.D. (1978). The epidemiology of breast cancer in 785 United States caucasian women. *Cancer*, **41**, 2341.